# Cell-wall properties contributing to improved deconstruction by alkaline pre-treatment and enzymatic hydrolysis in diverse maize (*Zea mays* L.) lines

**DOI:** 10.1093/jxb/erv016

**Published:** 2015-02-20

**Authors:** Muyang Li, Marlies Heckwolf, Jacob D. Crowe, Daniel L. Williams, Timothy D. Magee, Shawn M. Kaeppler, Natalia de Leon, David B. Hodge

**Affiliations:** ^1^Department of Biosystems & Agricultural Engineering, Michigan State University, East Lansing, MI 48824, USA; ^2^DOE-Great Lakes Bioenergy Research Center, 1552 University Ave., Madison, WI 53703, USA; ^3^Department of Chemical Engineering & Materials Science, Michigan State University, East Lansing, MI 48824, USA; ^4^Department of Agronomy, University of Wisconsin-Madison, Madison, WI 53706-1597, USA; ^5^Division of Sustainable Process Engineering, Luleå University of Technology, Luleå, Sweden 97187

**Keywords:** Biofuels, cell-wall recalcitrance, enzymatic hydrolysis, maize, plant cell-wall characterization, pre-treatment.

## Abstract

A maize (*Zea mays* L. subsp. *mays*) diversity panel consisting of 26 maize lines exhibiting a wide range of cell-wall properties and responses to hydrolysis by cellulolytic enzymes was employed to investigate the relationship between cell-wall properties, cell-wall responses to mild NaOH pre-treatment, and enzymatic hydrolysis yields. Enzymatic hydrolysis of the cellulose in the untreated maize was found to be positively correlated with the water retention value, which is a measure of cell-wall susceptibility to swelling. It was also positively correlated with the lignin syringyl/guaiacyl ratio and negatively correlated with the initial cell-wall lignin, xylan, acetate, and *p*-coumaric acid (*p*CA) content, as well as *p*CA released from the cell wall by pre-treatment. The hydrolysis yield following pre-treatment exhibited statistically significant negative correlations to the lignin content after pre-treatment and positive correlations to the solubilized ferulic acid and *p*CA. Several unanticipated results were observed, including a positive correlation between initial lignin and acetate content, lack of correlation between acetate content and initial xylan content, and negative correlation between each of these three variables to the hydrolysis yields for untreated maize. Another surprising result was that *p*CA release was negatively correlated with hydrolysis yields for untreated maize and, along with ferulic acid release, was positively correlated with the pre-treated maize hydrolysis yields. This indicates that these properties that may negatively contribute to the recalcitrance in untreated cell walls may positively contribute to their deconstruction by alkaline pre-treatment.

## Highlight

This work investigated the relationship between cell-wall properties in diverse maize lines that contribute to the differences in enzymatic hydrolysis yields for both untreated and mild NaOH-pre-treated biomass.

## Introduction

Structural polymers within plant cell walls (i.e. cellulose, hemicelluloses, and lignins) offer potential for long-term sustainable production of renewable fuels, chemicals, polymers, and materials that are currently produced from petrochemicals. Many of the promising conversion pathways for these products are based on cascades of biochemical and/or catalytic reactions starting with the sugars derived from cellulose (glucose) and hemicelluloses (primarily xylose in angiosperms). The recalcitrance of plant cell walls to biological degradation, deconstruction, or conversion is considered to be the most crucial factor to overcome in order to develop successful bioprocessing technologies for lignocellulose conversion to renewable fuels and chemicals (Himmel *et al.*, 2007). As such, in order to generate high sugar yields from the cellulose and hemicellulose within plant cell walls, pre-treatment is required in combination with the subsequent polysaccharide hydrolysis by either enzymes or an acid catalyst (Wyman *et al.*, 2011).

The grasses or graminaceous monocots represent many important agricultural residues such as corn (maize) stover (*Zea mays* L. subsp. *mays*), wheat straw (*Triticum* spp.), rice straw (*Oryza sativa* L.), and sugarcane bagasse (*Saccharum* spp.), as well as many promising perennial bioenergy feedstocks such as switchgrass (*Panicum virgatum* L.) and *Miscanthus* spp. (Vermerris, 2011). Outcomes of pre-treatment are obviously dependent on the combination of pre-treatment chemistry and the plant cell-wall properties. The cell walls of graminaceous monocots are known to contain alkali-labile ferulate ester cross-links within the hemicellulose and lignin (Scalbert *et al.*, 1985; Hatfield *et al.*, 1999; Iiyama and Lam, 2001), as well as high phenolic hydroxyl contents in their lignins, resulting in increased alkali solubility (Lapierre *et al.*, 1989), with these properties rendering the cell wall highly susceptible to delignification by alkaline pre-treatments (Li *et al.*, 2012; Liu *et al.*, 2014). As a consequence, mild alkali pre-treatment of grasses such as maize has shown substantial promise, as these can be employed for both fractionating biomass and generating a pre-treated biomass that is highly amenable to enzymatic hydrolysis (Chen *et al.*, 2013; Karp *et al.*, 2014; Liu *et al.*, 2014).

Corn stover (i.e. the stems and leaves) is considered the most promising agricultural residue in the USA for extensive deployment of technologies for biomass conversion to biofuels and biochemicals (US Department of Energy, 2011). A substantial body of literature exists on the correlation of cell-wall properties in maize with *in vitro* digestibility by rumen microbiota (Jung and Buxtono, 1994; Lundvall *et al.*, 1994; Argillier *et al.*, 2000; Jung *et al.*, 2000; Méchin *et al.*, 2000; Fontaine *et al.*, 2003; Lorenz *et al.*, 2010; Zhang *et al.*, 2011) and *in vivo* ruminant digestibility (Jung *et al.*, 1997), as well as hydrolysis by cellulolytic enzymes (Oakey *et al.*, 2013), with trends typically identifying strong negative correlations between the lignin content and compositions with cell-wall biological degradability. Other important initial cell-wall properties that have been correlated with digestibility or hydrolysis include the lignin syringyl/guaiacyl (S/G) ratio, lignin β-*O*-4 content, etherified ferulic acid (FA) content, and esterified *p*-coumaric acid (*p*CA) content (Jung and Buxtono, 1994; Méchin *et al.*, 2000; Iiyama and Lam, 2001). Strategies for engineering maize lines for its cellulosic biofuel or silage/feed value have been reviewed recently (Barrière *et al.*, 2003; Jung *et al.*, 2012) with strategies that include increasing the non-starch biomass yield and the total carbohydrate content (Vermerris *et al.*, 2007; de Leon *et al.*, 2013) and redirection of carbon to non-cellulosic sugars in the cell wall (e.g. β-glucan, starch; Slewinski, 2012; Chuck *et al.*, 2011; Chuck, 2013; Pauly *et al.*, 2014), as well as reducing the overall recalcitrance through decreasing the lignin content (He *et al.*, 2003), altering the lignin monomer content (Piquemal *et al.*, 2002), and alteration of feruloylation (Jung and Phillips, 2010; Barros-Rios *et al.*, 2012).

Cell-wall structural differences are typically neglected in cell-wall property correlation studies, although it is known that substantial differences in digestibility or sugar hydrolysis yields are found between cell types in untreated (Wilson *et al.*, 1993; Wilson and Hatfield, 1997; Hansey *et al.*, 2010) or pre-treated (Zeng *et al.*, 2012) grasses. As an example, Sarath *et al.* (2011) found that while cell-wall lignin content in diverse switchgrass lines was negatively correlated with hydrolysis yields following dilute acid pre-treatment, more than 50% of the variability could be attributed to differences in cell-wall architecture. The water retention value (WRV) has been used in our recent work as a predictor of enzymatic hydrolysis yields for corn stover and switchgrass subjected to alkali, alkaline oxidative, and liquid hot water pre-treatments (Williams and Hodge, 2014), and is proposed to act as an indirect measurement of a number of cell-wall properties (e.g. overall hydrophilicity, porosity, and polysaccharide accessibility) that are manifested in the cell wall’s ability to swell in water. This measurement may be able to incorporate structural differences between different cell-wall types such as the highly hygroscopic and digestible pith parenchyma cell walls versus the more hydrophobic and less-digestible cell walls within vascular bundles and the epidermis.

There is substantially less published literature on the correlation between plant cell-wall properties and their response to pre-treatment and hydrolysis. Recently, approaches for high-throughput screening of pre-treatment and enzymatic hydrolysis have been developed for the purpose of screening large sets of cell-wall material for the purpose of identifying potential reduced recalcitrance phenotypes both with and without a pre-treatment (Decker *et al.*, 2009; Santoro *et al.*, 2010). Understanding the cell-wall traits associated with reduced recalcitrance phenotypes or phenotypes that exhibit specific responses to pre-treatment are an important component of screening. However, typically only composition analysis is performed across the sample sets, limiting the understanding of the cell-wall properties, traits, or phenotypes that are also associated with the cell wall’s response to enzymatic hydrolysis. Notably, recent work has employed high-throughput screening of panels of promising bioenergy grasses that include diverse cultivars of wheat (*Triticum aestivum* L.) (Lindedam *et al.*, 2012), *Miscanthus* spp. (Zhang *et al.*, 2012), and maize (Penning *et al.*, 2014) subjected to hydrothermal pre-treatment, and a sorghum [*Sorghum bicolor* (L.) Moench] diversity panel subjected to aqueous ammonia pre-treatment (Vandenbrink *et al.*, 2010). Importantly, from these cited studies, while they were able to identify substantial differences in cell-wall responses to pre-treatment, it was difficult to draw conclusions about the cell-wall properties, traits, or phenotypes that are also associated with improved hydrolysis yields or sugar release following pre-treatment. Relative to other high-throughput screening studies of poplar, where lignin content as well as the S/G ratio could be clearly be identified as a strong contributor to hydrolysis yields following pre-treatment (Studer *et al.*, 2011), lignin was not correlated with hydrolysis yields in these studies, indicating that other unquantified cell-wall properties may be responsible for the observed variability in the data sets.

While at least one study has investigated the alteration of cell-wall properties and *in vitro* digestibility in a diverse set of orchard grass (*Dactylis glomerata* L.) subjected to acid chlorite delignification (Darcy and Belyea, 1980), there is scant literature on the relationship between plant cell-wall properties and hydrolysis yields for diverse maize lines subjected to delignifying pre-treatments such as mild NaOH pre-treatment. Thus, the goal of this work was to understand better how maize cell-wall properties impact initial recalcitrance as well as NaOH pre-treatment. Specifically, for this work 12 cell-wall properties were selected that may influence overall cell-wall recalcitrance including WRV, xylan and lignin contents before and after pre-treatment, initial cell-wall acetate content, *p*CA and FA content before pre-treatment, solubilized *p*CA and FA during pre-treatment, and the S/G ratio of the lignin in the untreated cell-wall. These properties were quantified for a diversity panel of 26 maize lines that were previously identified as exhibiting substantial phenotypic diversity in glucose release following mild NaOH pre-treatment (Muttoni *et al.*, 2012). These were subjected to mild NaOH pre-treatment and the glucose yields determined for the different lines before and after the pre-treatment. Subsequently, correlations between the cell-wall properties and hydrolysis yields were investigated.

## Materials and methods

### Maize diversity panel

The maize diversity set was grown in Arlington, WI, USA, in 2012 and the complete list of maize lines with accession number or resources is presented in Supplementary Table S1 at *JXB* online. The maize was harvested at grain physiological maturity with a Case IH^®^ 2144 axial-flow combine, which allows harvesting grain and biomass in a single pass as well as measuring whole-stover and grain weight and lastly chopping the whole stover. A subsample of approximately 1kg per plot was obtained and dried at 50 °C. The dried material was ground to a particle size of 1mm.

### NaOH pre-treatment, composition analysis, and enzymatic hydrolysis

Biomass samples were subjected to a mild alkaline pre-treatment whereby 2g of biomass was added to 20ml of an aqueous 8g l^–1^ NaOH solution in a 50ml centrifuge tube corresponding to an alkali loading of 0.08g NaOH g^–1^ of biomass. These tubes were placed in a static water bath at 80 °C for 1h. Following pre-treatment, the liquid was removed by filtration and the residual biomass was washed with deionized water until neutral. The pre-treatment mass yield was determined by quantifying the difference between the original and air-dried pre-treated materials gravimetrically. Composition analysis was carried out according to the NREL/TP 510–42618 to determine the structural carbohydrates, acetate, and Klason lignin of the untreated and pre-treated maize lines using a high-performance liquid chromatography (HPLC) machine equipped with an Aminex HPX-87 H (Bio-Rad, Hercules, CA, USA) column. The enzymatic hydrolysis was performed at pH 5.0, 50 °C, and 180rpm shaking, with a 30mg g^–1^ of protein loading of Cellic CTec2 (Novozymes A/S, Bagsværd, Denmark) on glucan (37.7 filter paper units g^–1^ glucan) for 6 or 72h. The yields were determined as the amount of glucose (as glucan) released following enzymatic hydrolysis divided by the glucan content of the untreated or pre-treated samples as determined by composition analysis. All data for cell-wall composition, pre-treatment mass yields, and hydrolysis yields are available in Supplementary Table S3 at *JXB* online. The composition analysis and enzymatic hydrolysis were performed in duplicate, with the data representing the mean and error bars representing the data range.

### Quantification of *p*-hydroxycinnamic acids

The *p*-hydroxycinnamic acids were determined by first treating 0.50g of biomass with 25ml of 3M NaOH in sealed pressure tubes at 120 °C for 1h in an autoclave to release both esterified *p*CA and FA and etherified FA. After cooling to room temperature, 250 μl of 10mg ml^–1^ *o*-coumaric acid in methanol was added as an internal standard for each sample. The mixture was transferred to 1.5ml centrifuge tubes and centrifuged at 13 000rpm for 10min. The pH of the supernatant was adjusted to 2.0 using concentrated HCl and the samples were then stored overnight at 4 °C. These samples were subsequently analysed by HPLC (Agilent 1100 Series) equipped with a C18 column (Discovery, 5 μm particle size, 5cm length by 2.1mm internal diameter; Sigma-Aldrich). Standards containing FA, *p*CA, and *o*-coumaric acid were also analysed and, together with the internal standard, were used to determine the concentration of the *p*-hydroxycinnamic acids in the samples. Concentrations were converted to a mass per original mass sample basis.

### Quantification of WRV

The WRV of untreated samples was determined as described in our previous work (Williams and Hodge, 2014). However, to avoid problems associated with sample drying for NaOH-pre-treated samples, 2g of biomass was freshly pre-treated with NaOH, filtered using a fabricated 200-mesh Buchner funnel, and rinsed with 500ml of deionized water. Subsequently, a plug of filtered, wet biomass was analysed as described in our previous work. Measurements were taken in triplicate.

### Determination of the S/G ratio

The S/G ratio was predicted by the combination of principal component analysis and partial least squares regression based on their pyrolysis molecular beam mass spectrometry (py-MBMS) spectra provided by Robert Sykes (National Renewable Energy Laboratory). The parameters of the prediction model were generated previously by correlating thioacidolysis S/G and py-MBMS spectra of a set of samples including untreated and alkaline oxidative pre-treated hybrid corn stover, brown midrib stover mutants *bm1* and *bm3*, switchgrass, and *Miscanthus*.

### Data analysis

Hierarchical cluster analysis according to Euclidian distance was performed in MATLAB (Mathworks, Natick, MA, USA) on the complete matrix of the Pearson’s correlation coefficients for all properties and yield combinations. Correlations were identified as significant if the *P* value associated with the proportionality coefficient was less than or equal to 0.05. Missing data points for some of the properties and yields were handled by only determining correlations and statistical parameters between sets of samples containing properties and yields that were being correlated.

## Results and discussion

### Cell-wall properties and hydrolysis yields

**Fig. 1.**
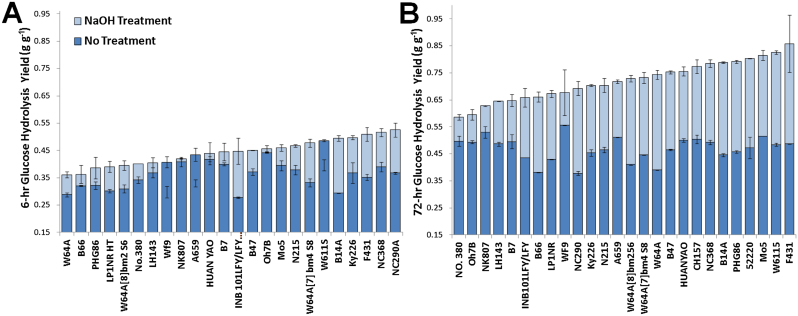
Range of hydrolysis yields obtained for untreated and NaOH-pre-treated maize for 6h (A) and 72h (B) hydrolysis yields. Error bars represent data range for duplicate samples. Due to missing data, some samples do not appear. (This figure is available in colour at *JXB* online.)

Twelve cell-wall properties or traits that may be indicators of cell-wall recalcitrance were quantified across the maize diversity set in addition to 6 and 72h hydrolysis yields prior to and following mild NaOH pre-treatment. The complete data set is available in Supplementary Table S3 with some of the important features describing the property data set presented in Table 1 and the yields presented in Fig. 1. As mentioned in the Introduction, WRV is proposed to act as a proxy variable that may be able to explain a number of phenomenon related to cell-wall polysaccharide accessibility to cellulolytic enzymes. As observed, untreated maize had WRVs ranging from approximately 1.9 to 2.4 with the WRV following alkaline treatment ranging from 2.3 to 3.3 (Table 1), clearly indicating a substantial increase in the cell-wall swelling and within the range for maize stover presented in our previous work (Williams and Hodge, 2014). Xylan concentration also exhibited substantial variability within the data set and increased in relative abundance from an average of 0.200 to 0.266g g^–1^ as a consequence of the removal of lignin and extractives. Wide ranges were observed for initial cell-wall acetyl content, *p*CA content, and FA content, as well as corresponding *p*CA and FA release. The S/G ratio as determined by py-MBMS coupled to partial least squares models also showed a diverse range, although within the range reported for maize (Morrison *et al.*, 1998).

**Table 1.** Variability within the data set for the 12 properties across the 27 maize lines

	Initial WRV (g g^–1^)	Final WRV (g g^–1^)	Initial xylan (g g^–1^)	Final xylan (g g^–1^)	Initial lignin (g g^–1^)	Final lignin (g g^–1^)	Initial acetate (mg g^–1^)	Initial *p*CA (mg g^–1^)	Initial FA (mg g^–1^)	*p*CA release (mg g^–1^)	FA release (mg g^–1^)	S/G ratio (mol mol^–1^)
	X_1_	X_2_	X_3_	X_4_	X_5_	X_6_	X_7_	X_8_	X_9_	X_10_	X_11_	X_12_
Min	1.7	2.2	0.16	0.20	0.13	0.068	26.7	8.7	9.0	4.1	4.4	0.68
Mean	2.0	2.7	0.20	0.27	0.17	0.11	33.1	11.9	11.3	5.9	5.7	0.97
Max	2.4	3.3	0.26	0.30	0.21	0.14	39.2	15.9	13.9	8.5	7.4	1.52
Std	0.17	0.30	0.02	0.02	0.02	0.02	3.3	1.7	1.3	1.3	0.79	0.20

Glucose hydrolysis yields ranged between 37.8 and 55.5% for untreated biomass and between 58.6 and 82.5% for NaOH-pre-treated biomass after 72h of hydrolysis (Fig. 1), clearly exhibiting a diverse range of digestibility phenotypes as well as diverse responses to mild NaOH pre-treatment. The 6h hydrolysis yields are intended to represent the initial hydrolysis rates, while the 72h hydrolysis yields represent the extent of hydrolysis as only minimal additional sugar release is observed beyond 72h (data not shown). It should be noted that these data awere plotted for glucose yield rather than glucose release, so that results were not biased towards cell walls with higher glucan content.

### Correlations between cell-wall properties

**Fig. 2.**
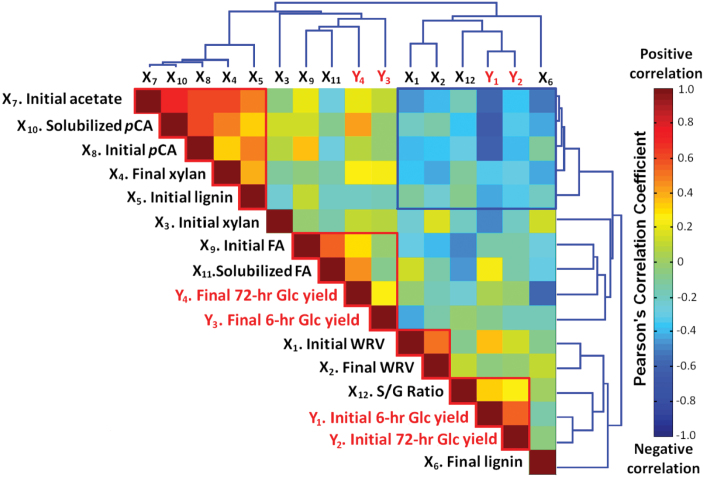
Correlation map of the Pearson’s correlation coefficients for the 12 cell-wall properties and four hydrolysis yields (red text) across the 27 maize lines as organized by hierarchical cluster analysis. Clusters of properties and yields exhibiting strong correlations are highlighted. ‘Initial’ indicates the property in the original untreated biomass sample, while ‘final’ indicates the property following pre-treatment.

**Fig. 3.**
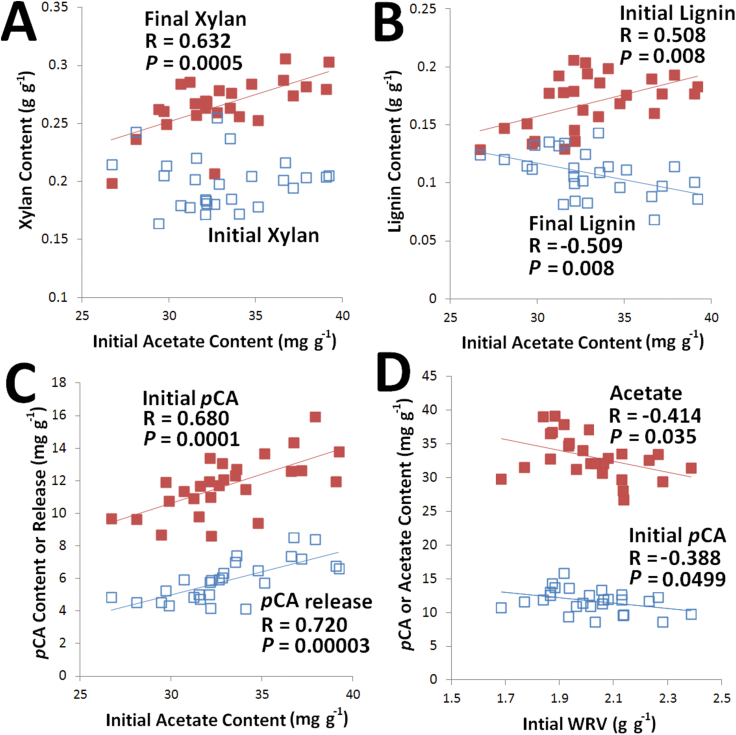
Several between-property correlations highlighted to demonstrate relationships within the data set. Each data point represents the property for one of the 27 maize lines. Pearson’s correlation coefficients and *P* values are presented for all property correlations with *P*≤0.05. Error bars on individual samples are not shown to improve clarity. (This figure is available in colour at *JXB* online.)

In order to visualize better the relationships between the variables, a correlation map was developed and organized using hierarchical clustering according to the Euclidian distance between sets of the Pearson correlation coefficients (*R*) (Fig. 2). The magnitude, scale, and significance of the correlations are presented in Supplementary Table S2 at *JXB* online. Within the correlation map (Fig. 2), several multi-property clusters of positive correlations were observed along the top left to bottom right diagonal, while one multi-property cluster of negative correlations stood out in both the bottom left and top right corners. This indicated that there a number of properties that are correlated across diverse maize lines and may be responsible for differences in the cell wall’s response to enzymatic hydrolysis as well as the response to pre-treatment. A number of strong positive correlations between related properties or yields were observable along the top left to bottom right diagonal, namely the initial 6 and final 72h hydrolysis yields, initial *p*CA content and *p*CA solubilization, initial FA content and FA solubilization, and initial and final WRV. These specific results are not surprising as they may be expected to be related. A number of property correlations that highlight either causal or merely correlative relationships between cell-wall properties (but not hydrolysis yields) from within the highlighted clusters were selected and replotted in Fig. 3. These correlations were plotted using either initial acetate content or initial WRV as the abscissa, as these properties appeared within two of the important clusters. It should be stressed that acetate content and WRV are not necessarily the properties responsible for the variations in the other properties but were merely correlated to them.

Xylans (as well as other non-cellulosic cell-wall polysaccharides) are known to be substantially *O*-acetylated and the role of these substitutions is hypothesized to be to controlling the strength of glycan–glycan cross-linking by preventing H-bonding between cell-wall polymers (Carpita, 1996; Gille and Pauly, 2012; Pawar *et al.*, 2013). Of note is that, while the acetate content showed a diverse range (26.7–39.2mg g^–1^), there was no correlation with the cell-wall xylan content (Fig. 3A), indicating either that the xylans exhibited either a wide range of *O*-acetylation—calculated to be 0.25–0.60 moles of acetyl per mole of xylopyranosyl, which is within the reported range for maize (Selig *et al.*, 2009)—or that the acetyl groups are substituted on moieties other than xylan (e.g. glucomannans, pectins, or lignins). While uncorrelated to the initial xylan content (Fig. 3A), this diverse range of acetate contents could be observed to exhibit correlations with a number of other cell-wall properties, including strong, statistically significant positive correlations with the final xylan content (Fig. 3A), the initial lignin content (Fig. 3B), and the initial content and release of *p*CA (Fig. 3C), while showing negative correlations to the initial lignin content (Fig. 3B) and a strong positive correlation to both the final lignin content (Fig. 3B) and the initial WRV (Fig. 3D).

One of the primary outcomes of alkaline pre-treatment of grasses is the saponification of ester bonds in the plant cell-wall biopolymers resulting in solubilization of acetate, FA, *p*CA, xylans, and lignin fragments. The acetate results can be interpreted as potentially relating to the lignin release. While it may be expected that high-acetate contents would consume more alkali and presumably hinder lignin removal, the opposite is the case. As observed in Fig. 3B, the initial cell-wall acetate content was positively correlated to initial lignin contents and negatively correlated to final lignin content, indicating that high-acetate cell walls are likely to be more recalcitrant yet respond better to alkaline pre-treatment with respect to lignin removal. Additionally, it was observed that initial *p*CA content and *p*CA released were positively correlated with initial acetate content (Fig. 3C). This may be a consequence of the correlation of between acetate and lignin since *p*CA is known to be acylated to syringyl lignins and may only represent the correlation to lignin content (Jung and Allen, 1995). The correlation to final xylan content may be a consequence of high acetate corresponding to higher lignin removal, which would enrich the xylan content of the pre-treated biomass. Overall, it is not clear whether this cluster of co-varying properties is an indication of differences in the ‘average’ cell-wall property or whether these may indicate differences in the abundance of, for example, high-lignin, high-*p*CA, and high-acetate tissues. Recently, work has been targeted at altering the expression of genes associated with *O*-acetylation as a strategy for altering cell-wall recalcitrance (Xiong *et al.*, 2013), although it is currently not clear how only alteration in xylan *O*-acetylation will impact cell-wall recalcitrance.

Interestingly, WRVs did not show a significant correlation with any of the cell-wall biopolymer content (i.e. lignin and xylan) while it did exhibit significant correlations (albeit weak) with substitutions on these biopolymers (i.e. *p*CA and acetate; Fig. 3D). Specifically, the initial cell-wall *p*CA content was inversely correlated to the initial WRV, which may provide evidence that highly *p*-coumaroylated cell walls may be more hydrophobic (and higher in lignin content), potentially imparting increased recalcitrance to degradation by microbial pathogens or rumen microbiota. The (weak) negative correlation between initial cell-wall acetate content and initial WRV (Fig. 3D) could potentially be due to increasing hydrophobicity of exposed xylans (i.e. the glycan will have an exposed ethyl group rather than a hydroxyl group). However, the hypothesis that highly acetylated cell walls are more porous due to a decreased level of H-bonding between xylans and cellulose does not fit this data since increasing acetyl content corresponded to decreasing WRVs (Fig. 3D). As initial lignin content was positivity correlated with the cell-wall acetate content (Fig. 3B), the decreasing initial WRV with increasing acetate could be a consequence of the increasing lignin content decreasing the cell-wall’s capacity to absorb water.

### Correlations between cell-wall properties and hydrolysis yields

**Fig. 4.**
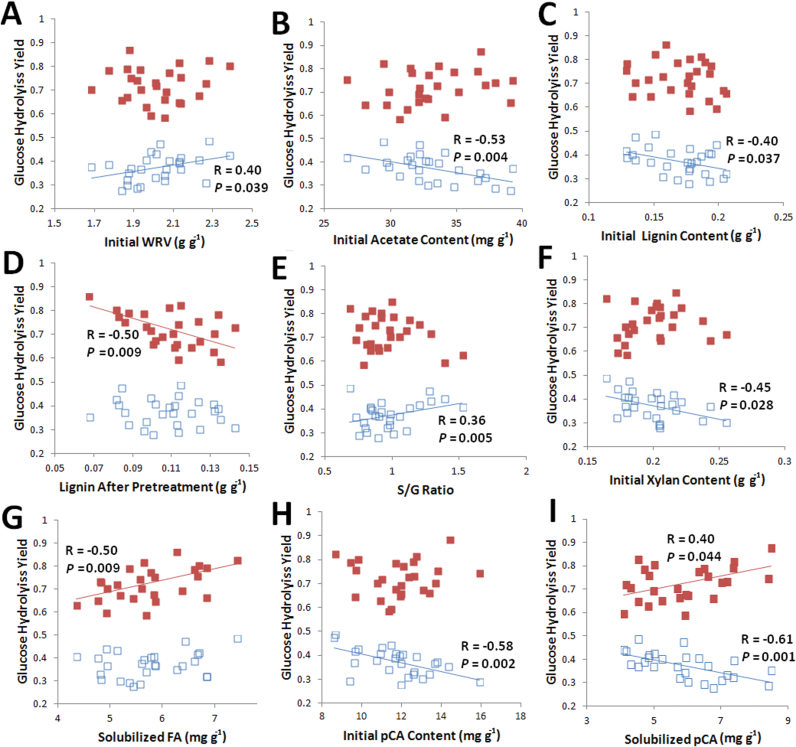
Summary of significant property correlations with glucose hydrolysis yields. Open data points represent 6h hydrolysis yields for untreated biomass; filled data points represent 72h hydrolysis yields for NaOH-pre-treated biomass. Each data point represents the value of the property and corresponding yield for one of the 27 maize lines. Pearson’s correlation coefficients and *P* values are presented for all property correlations with *P*≤0.05. Error bars on individual samples are not shown to improve clarity. (This figure is available in colour at *JXB* online.)

Besides between-property correlations, correlations between cell-wall properties and hydrolysis yields are important for understanding property contributions to cell-wall recalcitrance. All significant (*P*≤0.05) correlations between cell-wall properties and hydrolysis yields are plotted in Fig. 4. Notably, this plots only shows correlations for untreated 6h yields and NaOH-pre-treated 72h yields. The untreated 72h yield did not exhibit significant correlations with any properties other than the untreated 6h yields (Fig. 2 and Table 1). However, the correlations that were strongest for the untreated 6h hydrolysis yields were not that significant for 72h digestibility, exhibiting similar correlations with *P* values between 0.05 and 0.15. The differences between the hydrolysis yields obtained at different time points (6 vs 72h) may indicate that the initial cell-wall composition impacts the hydrolysis rate more strongly than the hydrolysis extent. The 6h hydrolysis yields for NaOH-pre-treated corn stover were not found to exhibit strong correlations with any other properties and, furthermore, were often lower than the untreated 6h hydrolysis yields (Fig. 1B). This contradictory result may be due to the drying of the pre-treated material necessitated by the analysis that resulted in its stronger resistance to rehydration, which may have introduced more variability in the data for the initial glucose release by hydrolysis but presumably not the extent of hydrolysis.

A number of important trends can be identified from the data in Fig. 4. The first is the identification that WRV was positively correlated with the untreated 6h hydrolysis yield (Fig. 4A). As discussed in the introduction, WRV was included as a parameter that may offer the potential for consolidating a number of other, unquantified cell-wall properties that may be able to be correlated with hydrolysis yields. Our previous work used only two types of biomass (corn stover and switchgrass) subjected to varying levels of delignification by alkaline oxidative pre-treatment and found strong correlations between WRV and hydrolysis yields (Williams and Hodge, 2014). The current work used a wide range of (similar) biomass that was subjected to a single pre-treatment condition, which may explain why WRVs following pre-treatment were not able to predict differences in hydrolysis yields following pre-treatment.

The initial cell-wall acetate content demonstrated a negative correlation with the untreated 6h hydrolysis yield although not with the pre-treated 72h yield (Fig. 4B). As discussed previously, the acetate content is strongly correlated to many other properties and potentially this cluster of related properties may be able to explain differences in hydrolysis yields rather than a single property. For example, initial acetate content was strongly correlated with initial lignin content (Fig. 3B), which may be the property most strongly responsible for the response in hydrolysis yields. Previously, non-linear models have been developed relating decreases in acetate and lignin to increases in hydrolysis yields for lime-pre-treated corn stover (Kim and Holtzapple, 2006) and hybrid poplar (O’Dwyer *et al.*, 2008), although these correlated simultaneous acetate and lignin removal by alkali with improved hydrolysis yields rather than initial variability in these properties, as was done in the present work.

Lignin is thought to impact saccharification yields by physical occlusion of polysaccharides (Brunecky *et al.*, 2009), providing resistance to swelling (O’Neil and York, 2003), and by non-specific binding to cellulases (Palonen *et al.*, 2004). Studies have generally shown a strong negative correlation between lignin content and hydrolysis yields for a wide range of untreated grasses (Jung and Vogel, 1986; Jung *et al.*, 1997; Zhang *et al.*, 2011) with strong correlations following a delignifying pre-treatment (Sewalt *et al.*, 1997; Kim and Holtzapple, 2006; Li *et al.*, 2012). As expected, the untreated lignin content was negatively correlated with the untreated 6h hydrolysis yield (Fig. 4C) and the lignin content following pre-treatment was negatively correlated with the pre-treated 72h hydrolysis yield (Fig. 4D). However, the lignin contents prior to and following pre-treatment did not correlatedwith each other (Fig. 2 and Table 1), indicating that lignin removal for mild NaOH pre-treatment is not necessarily dependent on the initial lignin content but is dependent on many other cell-wall properties. This is significant in that many strategies for reduced cell-wall recalcitrance have targeted lignin levels (Simmons *et al.*, 2010), although it may be sufficient that the lignin is easily removable by the pre-treatment to decrease the final cell-wall recalcitrance rather than the initial cell-wall recalcitrance.

As we have already identified in this work, the lignin removal is more important than the initial lignin content for mild NaOH pre-treatment and consequently, lignin properties that may contribute to improved lignin removal are important. Another interesting lignin-related finding was that the initial S/G ratio was positively correlated with the untreated 6h hydrolysis yield (Fig. 4E), while the trend apparently reversed for the pre-treated hydrolysis yield (although this was not a statistically significant correlation). The correlation between S/G ratio in untreated biomass and *in vitro* ruminant digestibility for grasses has been somewhat contradictory in the literature (Méchin *et al.*, 2000, 2005; Zhang *et al.*, 2011) although a general trend is that an increasing S/G ratio may be linked to increasing digestibility.

The initial xylan content was found to be negatively correlated with the untreated 6h hydrolysis yield (Fig 4F). The fact that both initial lignin and xylan were negatively correlated with the untreated hydrolysis yields yet these two properties were uncorrelated with each (Fig. 2) may indicate that both of these components play an important role in the limited access of cellulolytic enzymes to cellulose. The enzyme cocktail utilized for this study was not supplemented with xylanase, which may be a factor contributing to this result.

Ferulate and diferulate esters are known to be attached to the primary hydroxyl at the C5 position of α-l-arabinofuranosyl residues in xylans and be involved in cross-linking of xylans to lignin by incorporation of the aromatic moiety through ether linkages into growing lignin polymers or by cross-coupling (Hatfield *et al.*, 1999). This cross-linking of cell-wall polymers by ferulates is generally accepted to play an important role in cell-wall recalcitrance (Grabber *et al.*, 1998). Negative correlations have been found in the literature for total cell-wall ferulate content and *in vitro* ruminant digestibility in maize (Jung *et al.*, 2011), as well as in other diverse grasses (Iiyama and Lam, 2001), and decreasing cell-wall ferulate content has been investigated as a strategy for improving the forage quality of maize (Jung and Phillips, 2010). However, while strong, statistically significant negative correlations were identified between etherified ferulates and *in vitro* digestibility, esterified ferulate content was found to be positively correlated with *in vitro* digestibility in smooth bromegrass (*Bromus inermis* Leyss subsp. inermis), cocksfoot (*D. glomerata* L.), and reed canary grass (*Phalaris arundinacea* L.) (Casler and Jung, 2006; Casler *et al.*, 2008), and other recent work has validated a strategy for increasing esterified diferulate content in maize to improve its digestibility (Barros-Rios *et al.*, 2012). The present work did not distinguish between etherified and esterified ferulates, although the method for ferulate content was performed under harsh enough conditions that this term probably comprises both etherified and esterifed as increasing the alkali loading and temperature during saponification did not result in additional ferulate release (data not shown). The ferulate release during pre-treatment may be more representative of esterified ferulate due to the mild conditions utilized for pre-treatment. Nonetheless, ferulate content and the ferulate released were found to be strongly positively correlated to each other (Fig. 2 and Table 1) and, importantly, the ferulate released showed a strong correlation with the 72h hydrolysis yields following NaOH pre-treatment (Fig. 4G), although not with any of the other hydrolysis conditions. This can be understood, as cell walls that are highly cross-linked are more susceptible to alkaline pre-treatment. Recently, incorporation of ferulate esters into hybrid poplar has been validated as a strategy to improve enzymatic hydrolysis following an alkaline pre-treatment (Wilkerson *et al.*, 2014).

Lignin content, *p*CA content, and the S/G ratio in maize stem internodes and rinds have been shown to increase with increasing maturity, while ferulate content does not change (Morrison *et al.*, 1998). The simultaneous increase in these properties has been implicated in the decrease in maize digestibility with increasing maturity (Grabber *et al.*, 2004; Jung and Casler, 2006). Both the initial *p*CA (Fig. 4H) and solubilized *p*CA (Fig. 4I) exhibited statistically significant correlations with the hydrolysis yields. These results were notable in that the correlation was the opposite for untreated biomass compared with NaOH-pre-treated biomass, whereby increasing release or content of *p*CA corresponded to decreasing hydrolysis yields for untreated cell walls and increasing hydrolysis yields for pre-treated cell walls. High *p*CA content in untreated maize has been correlated with low *in vitro* digestibility (Méchin *et al.*, 2000; Zhang *et al.*, 2011), although the finding that high initial *p*-coumarylation of lignin may be related to high hydrolysis yields following pre-treatment is novel. As discussed previously, the *p*CA content was positively correlated with the initial lignin content, so this could be an indirect measure of the impact of lignin content. As another alternative, *p*CA-containing lignins may be responsible for the uptake and inactivation of cellulolytic enzymes, as is known to occur for polyphenolic compounds such as tannins and lignins and phenolic acid monomers (Ximenes *et al.*, 2011).

## Conclusions

Understanding the relationship between cell-wall recalcitrance and pre-treatment is important in that this may lead to the identification of strategies for plant breeding or genetic engineering that may improve cell-wall deconstruction. For this study, 26 diverse maize lines were subjected to mild NaOH pre-treatment and a broad set of properties relating to cell-wall recalcitrance were characterized. By combining cell-wall structural characterization and enzymatic hydrolysis of cell-wall polysaccharides both prior to and following mild NaOH pre-treatment, a number of expected as well as non-intuitive results were identified. The hydrolysis yields of non-pre-treated cell walls were found to be positively correlated with WRV and S/G ratio and negatively correlated with xylan and acetate content. While the initial cell-wall xylan and acetate content were uncorrelated with each other, the acetate content was found to exhibit a number of strong correlations with other cell-wall properties. The pre-treated cell-wall hydrolysis yields were positively correlated with the ferulate released by pre-treatment, indicating that breaking of ferulate cross-links between cell-wall polymers is an important outcome of pre-treatment. As expected, statistically significant negative correlations were identified between the cell-wall lignin content and the hydrolysis yields for both untreated (*R*=–0.40, *P*=0.037) and NaOH-pre-treated maize (R=–0.50, *P*=0.009). It has long been known that cell-wall lignin content can be negatively correlated with enzymatic hydrolysis yields and *in vitro* digestibility, as well as *in vivo* digestibility in ruminants. However, the data demonstrated that the initial cell-wall lignin content and the pre-treated cell-wall content were not correlated with each other, that initial cell-wall lignin content was not correlated with hydrolysis yields following pre-treatment, and the cell-wall lignin content following pre-treatment was not correlated with untreated hydrolysis yields. This important finding indicates that, while enzymatic hydrolysis yields may be set by the cell-wall lignin content, the cell wall’s response to delignifying pre-treatment such as mild NaOH pre-treatment is not necessarily set by the initial lignin content. The *p*CA that was saponifiable by mild NaOH pre-treatment showed a negative correlation with the hydrolysis yields of untreated maize (*R*=–0.61, *P*=0.001) and the inverse response for pre-treated maize (*R*=0.40, *P*=0.044), indicating that cell walls with a high content of saponifiable *p*CA are more recalcitrant without treatment, yet respond better to pre-treatment than cell walls with a low content of saponifiable *p*CA. Importantly, this indicates that properties contributing to a ‘reduced recalcitrance’ phenotype following a specific pre-treatment are not necessarily the same properties that contribute to recalcitrance in untreated cell walls.

## Supplementary data

Supplementary data are available at *JXB* online.

Supplementary Table S1. Genotype and reference source for the 27 maize lines used in this work.

Supplementary Table S2. Calculated proportionality constants (unscaled) between all cell-wall properties and hydrolysis yields.

Supplementary Table S3. Complete data set for cell-wall composition prior to and following pre-treatment, mass yields following pre-treatments, quantified properties, and hydrolysis yields.

Supplementary Data

## Abbreviations:

FA: ferulic acid
HPLC: high-performance liquid chromatography
*p*CA: *p*-coumaric acid
py-MBMS: pyrolysis molecular beam mass spectrometry
S/G: syringyl/guaiacyl
WRV: water retention value.
