# Supplementary material for: Cell-wall properties contributing to improved deconstruction by alkaline pre-treatment and enzymatic hydrolysis in diverse maize (Zea mays L.) lines
Source: J Exp Bot. 2015 Feb 20;66(14):4305–15. doi: 10.1093/jxb/erv016 (PMC4493778; doi:10.1093/jxb/erv016)
Supplement: Supplementary Data [file supp_66_14_4305__index.html]

Cell-wall properties contributing to improved deconstruction by alkaline pre-treatment and enzymatic hydrolysis in diverse maize (Zea mays L.) lines — Supplementary Data 

# Cell-wall properties contributing to improved deconstruction by alkaline pre-treatment and enzymatic hydrolysis in diverse maize (*Zea mays* L.) lines

## Supplementary Data

Data files

- Supplementary Data - Supplementary Data
- Supplementary Data - Supplementary Data
