# Supplementary material for: Cell-wall properties contributing to improved deconstruction by alkaline pre-treatment and enzymatic hydrolysis in diverse maize (Zea mays L.) lines
Source: J Exp Bot. 2015 Feb 20;66(14):4305–15. doi: 10.1093/jxb/erv016 (PMC4493778; doi:10.1093/jxb/erv016)
Supplement: Supplementary Data [file supp_erv016_jexbot134593_file002.pdf]

Supplemental Table S1: Genotype and reference source for the 27 maize lines used in this work.

Genotype	Accession Number or Source
52220	Ames 2336
A659	NSL 81599
B14A	PI 550461
B47	PI 601009
B66	PI 550464
B7	NSL 65863
CH157	Ames 22441
F431	PI 257515
HUANYAO	Ames 2337
INB 101LFY/LFY (A632 X M16 S5)	NSL 174429
Ky226	Ames 27131
LH143 (Maintainer)	Ames 27450
LP1NR HAT	PI 600729
Mo5	PI 558523
N215	PI 595367
NC290A	Ames 27141
NC368	Ames 27180
NK807	PI 601430
NO. 380	PI 303924
Oh7B	Ames 19323
PHG86	PI 601442
W611S	[a]
W64A	NSL 30058
W64A[7] bm4 S8	[a]
W64A[8]bm2 S6	[a]
Wf9	Ames 19293
YE 4	Ames 2335

[a] Dr. Natalia de Leon - University of Wisconsin (ndeleongatti@wisc.edu)

Supplemental Table S2. Calculated proportionality constants (unscaled) between all cell wall properties and hydrolysis yields. * indicates the parameter is significant at $p \leq 0.05$. ** indicates the parameter is significant at $p \leq 0.01$.

	X_1	X_2	X_3	X_4	X_5	X_6	X_7	X_8	X_9	X_{10}	X_{11}	X_{12}	Y_1	Y_2	Y_3	Y_4
X_1	1	0.54**	-0.21	-0.33	-0.06	-0.04	-0.41*	-0.37*	-0.26	-0.16	0.19	-0.06	0.40*	0.18	-0.40	0.06
X_2		1.00	0.19	-0.40	-0.20	0.15	-0.37	-0.33	-0.36	-0.12	-0.10	0.13	0.00	0.00	-0.11	-0.12
X_3			1.00	-0.09	-0.20	0.19	-0.03	0.12	0.02	0.18	-0.12	-0.18	-0.45*	-0.17	0.10	0.12
X_4				1.00	0.43*	-0.39	0.63**	0.37	-0.02	0.52	-0.11	-0.08	-0.31	-0.26	0.27	0.24
X_5					1.00	-0.16	0.50**	0.52	0.15	0.36	-0.18	-0.06	-0.40*	-0.30	-0.15	-0.18
X_6						1.00	-0.48**	-0.09	-0.25	-0.39	-0.27	0.05	-0.11	-0.03	-0.13	-0.50**
X_7							1.00	0.65**	0.24	0.73**	-0.22	-0.14	-0.53**	-0.34	0.13	0.20
X_8								1.00	0.38	0.65**	-0.25	-0.26	-0.58**	-0.35	0.00	0.03
X_9									1.00	0.16	0.59**	-0.44	-0.11	-0.12	0.02	0.32
X_{10}										1.00	-0.05	-0.32	-0.61**	-0.30	0.02	0.40*
X_{11}											1.00	-0.41*	0.25	-0.11	-0.03	0.50**
X_{12}												1.00	0.36**	0.31	0.03	-0.24
Y_1													1.00	0.59**	-0.05	0.10
Y_2														1.00	-0.15	0.01
Y_3															1.00	0.25
Y_4																1.00

X_1: Initial WRV
X_2: Final WRV
X_3: Initial Xylan
X_4: Final Xylan
X_5: Initial Lignin
X_6: Final Lignin
X_7: Initial Acetate
X_8: Initial *p*CA
X_9: Initial FA
X_{10}: *p*CA release
X_{11}: FA release
X_{12}: S/G ratio
Y_1: Initial 6-hr Glc yield
Y_2: Initial 72-hr Glc yield
Y_3: Final 6-hr Glc yield
Y_4: Final 72-hr Glc yield

Supplemental Table S3: Complete data set for cell wall composition prior and following pretreatment, mass yields following pretreatments, quantified properties, and hydrolysis yields.

Name	Initial WRV (g g^{-1}) X1	Final WRV (g g^{-1}) X2	Initial Xylan (g g^{-1}) X3	Final Xylan (g g^{-1}) X4	Initial Lignin (g g^{-1}) X5	Final Lignin (g g^{-1}) X6	Initial Acetate (g mg^{-1}) X7	Initial pCA (g mg^{-1}) X8	Initial FA (g mg^{-1}) X9	pCA release (g mg^{-1}) X10	FA release (g mg^{-1}) X11	S/G Ratio X12	Untreated 6-hr Yield Y1	Untreated 72-hr Yield Y2	Pretreated 6-hr Yield Y3	Pretreated 72-hr Yield Y4
52220	2.387	3.008	0.202	0.267	0.178	0.081	31.491	9.792	11.603	5.002	6.704	0.912	0.426	0.473		0.804
A659	2.015	2.659	0.184	0.267	0.146	0.099	32.147	10.987	10.856	4.165	5.132	1.284	0.434	0.511	0.330	0.718
B14A	1.934	2.695	0.205	0.284	0.168	0.096	34.753	9.402	9.057	6.484	5.360	1.002	0.294	0.446	0.494	0.788
B47	1.884	2.385	0.205	0.303	0.183	0.086	39.211	13.789	11.753	6.601	5.842	0.866	0.372	0.465	0.450	0.753
B66	2.058	2.708	0.172	0.263	0.206	0.113	32.099	13.384	11.967	5.753	6.850	0.961	0.320	0.381	0.363	0.661
B7	2.130	2.666	0.205	0.261	0.134	0.112	29.692	11.899	9.721	5.225	4.768	0.833	0.400	0.495	0.445	0.648
CH157	2.082	2.630	0.198	0.279	0.194	0.083	32.865	12.060	11.693	6.323	5.779	1.122	0.403	0.504		0.774
F431	1.874	2.511	0.217	0.306	0.160	0.068	36.709	14.340	12.314	8.486	6.279	0.978	0.352	0.487	0.509	0.858
HUAN YAO	2.139	3.149	0.215	0.198	0.129	0.124	26.693	9.664	11.820	4.834	6.666	1.197	0.418	0.501	0.439	0.756
INB 101LFY/LFY (A632xM16S5)	1.841	2.376	0.204	0.280	0.177	0.100	39.041	11.951	12.140	6.767	5.433	0.889	0.278	0.436	0.448	0.658
Ky226	1.936	2.359	0.178	0.252	0.176	0.111	35.112	13.666	10.736	5.695	4.936	1.048	0.368	0.454	0.497	0.703
LH143	2.137	3.022	0.243	0.236	0.147	0.120	28.082	9.626	10.519	4.519	5.857	0.896	0.369	0.487	0.405	0.646
LP1NR HT	1.867	2.500	0.255	0.259	0.204	0.125	32.778	13.061	11.434	6.012	5.188	0.802	0.302	0.430	0.390	0.672
Mo5	2.132	2.549	0.185	0.276	0.186	0.109	33.586	12.717	10.998	7.371	5.643	0.846	0.395	0.515	0.460	0.815
N215	1.686	2.767	0.214	0.249	0.136	0.132	29.866	10.737	10.799	4.299	5.594	0.980	0.379	0.465	0.468	0.704
NC290A	2.063	2.821	0.185	0.270	0.179	0.105	32.088	11.946	12.874	4.989	6.379	0.726	0.367	0.378	0.525	0.692
NC368	1.772	2.234	0.221	0.257	0.129	0.134	31.563	11.672	13.857	4.708	6.616	0.912	0.389	0.492	0.516	0.785
NK807	1.962	2.552	0.178	0.286	0.192	0.132	31.217	10.927	9.382	4.819	4.359	1.522	0.408	0.530	0.419	0.628
No.380	2.058	2.842	0.180	0.284	0.177	0.135	30.661	11.347	10.392	5.926	5.691	0.785	0.342	0.497	0.402	0.586
Oh7B	1.986	2.573	0.172	0.256	0.199	0.114	34.058	11.445	11.018	4.101	4.920	1.384	0.442	0.494	0.457	0.595
PHG86	1.868	2.540	0.202	0.287	0.190	0.088	36.571	12.569	13.791	7.350	6.847	0.795	0.322	0.457	0.386	0.792
W611S	2.282	2.437	0.164	0.262	0.151	0.115	29.415	8.657	11.307	4.526	7.433	0.682	0.486	0.484	0.395	0.825
W64A	1.919	2.223	0.204	0.282	0.193	0.114	37.877	15.914	12.536	8.404	5.572	0.749	0.288	0.391	0.361	0.743
W64A[7] bm4 S8	2.012	3.321	0.195	0.274	0.177	0.097	37.159	12.607	10.735	7.181	4.807	0.978	0.333	0.446	0.477	0.732
W64A[8]bm2 S6	2.266	3.321	0.237	0.263	0.157	0.143	33.515	12.322	9.022	7.009	4.816	1.099	0.309	0.411	0.395	0.730
Wf9	2.231	2.873	0.181	0.207	0.163	0.102	32.604	11.687	12.271	5.931	5.833	0.835	0.407	0.555	0.298	0.677
YE4	2.032	2.581	0.181	0.269	0.136	0.084	32.171	8.594	9.796	5.881	6.435	1.272	0.474	0.452		

	Error +/-	Error +/-	Error +/-	Error +/-	Error +/-	Error +/-	Error +/-	Error +/-	Error +/-	Error +/-	Error +/-	Error +/-	Error +/-	Error +/-	Error +/-	Error +/-
52220	0.045	0.137	0.0070	0.0027	0.0008	0.0030	0.3594	0.573	0.388	0.299	0.218	0.314	0.037	0.040		0.001
A659	0.062	0.148	0.0013	0.0026	0.0017	0.0097	0.3979	0.300	0.131	0.165	0.228	0.067	0.024	0.000	0.013	0.006
B14A	0.085	0.069	0.0038	0.0007	0.0073	0.0039	0.1307	1.133	1.388	0.583	0.493	0.027		0.005	0.010	0.002
B47	0.061	0.062	0.0038	0.0014	0.0020	0.0055	0.7081	0.604	0.277	0.351	0.244	0.116	0.012	0.002		0.006
B66	0.217	0.140	0.0013	0.0002	0.0020	0.0094	0.6794	0.132	0.122	0.207	0.365	0.095	0.002		0.034	0.019
B7	0.031	0.256	0.0180	0.0015	0.0033	0.0016	0.4809	0.579	0.342	0.230	0.300	0.032	0.006	0.027	0.032	0.023
CH157	0.063	0.038	0.0003	0.0000	0.0018	0.0016	0.1461	0.365	0.158	0.272	0.179	0.253	0.003	0.016		0.026
F431	0.091	0.187	0.0001	0.0191	0.0033	0.0061	1.0413	0.638	0.983	0.224	0.139	0.231	0.010	0.001	0.025	0.106
HUAN YAO	0.100	0.201	0.0100	0.0132	0.0028	0.0003	1.2652	0.887	0.885	0.386	0.345	0.031	0.009	0.006	0.041	0.017
INB 101LFY/LFY (A632xM16S5)	0.100	0.047	0.0016	0.0005	0.0081	0.0076	1.0073	0.536	0.263	1.274	1.210	0.191	0.003	0.000	0.048	0.034
Ky226	0.117	0.227	0.0010	0.0021	0.0050	0.0061	1.1164	0.246	0.100	0.200	0.141	0.005	0.037	0.011	0.008	0.004
LH143	0.058	0.336	0.0044	0.0150	0.0000	0.0229	0.5002	0.528	0.616	0.336	0.403	0.091	0.019	0.008	0.018	0.001
LP1NR HT	0.228	0.128	0.0011	0.0071	0.0009	0.0101	0.0511	1.103	0.992	0.585	0.531	0.185	0.006	0.000	0.020	0.012
Mo5	0.057	0.065	0.0068	0.0026	0.0005	0.0056	0.0182	0.209	0.072	0.344	0.171	0.317	0.019		0.012	0.019
N215		0.092	0.0027	0.0015	0.0159	0.0233	0.1943	0.263	0.092	0.079	0.263	0.031	0.017	0.010	0.005	0.026
NC290A	0.065	0.120	0.0001	0.0117	0.0036	0.0050	0.8118	0.250	0.329	0.694	0.937	0.054	0.003	0.008	0.026	0.026
NC368	0.082	0.113	0.0015	0.0000	0.0116	0.0000	0.9404	0.185	0.400	0.527	0.614	0.080	0.018	0.008	0.015	0.014
NK807	0.036	0.039	0.0010	0.0031	0.0049	0.0066	0.4493	1.244	1.145	0.106	0.110	0.074	0.017	0.021	0.003	0.002
No.380	0.024	0.137	0.0044	0.0084	0.0032	0.0085	0.2223	0.297	0.315	0.343	0.306	0.018	0.011	0.018		0.011
Oh7B	0.227	0.304	0.0048	0.0005	0.0060	0.0125	0.0178	0.165	0.149	0.304	0.245	0.211	0.002	0.006	0.012	0.020
PHG86	0.064	0.204	0.0024	0.0130	0.0068	0.0084	0.3983	0.137	0.259	0.517	0.212	0.119	0.014	0.005	0.039	0.005
W611S	0.151	0.314	0.0070	0.0004	0.0020	0.0196	0.8643	0.324	0.167	0.509	0.355	0.417	0.003	0.005	0.020	0.003
W64A	0.036	0.088	0.0004	0.0038	0.0014	0.0094	0.6330	0.201	0.181	0.234	0.295	0.055	0.007	0.001	0.011	0.017
W64A[7] bm4 S8	0.041	0.180	0.0051	0.0195	0.0009	0.0039	0.8313	0.208	0.148	0.612	0.207	0.112	0.015	0.001	0.015	0.020
W64A[8]bm2 S6		0.263	0.0097	0.0174	0.0058	0.0086	1.0368	0.388	0.326	0.309	0.189	0.154	0.015	0.002	0.017	0.012
Wf9	0.122	0.273	0.0021	0.0256	0.0033	0.0078	1.0512	0.093	0.095	0.268	0.153	0.119	0.021	0.001	0.020	0.085
YE4	0.090	0.190	0.0014	0.0093	0.0124	0.0098	0.4222	0.910	0.928	0.244	0.336	0.189	0.018		0.025	
Replicates:	N=3	N=3	N=2	N=2	N=2	N=2	N=2	N=4	N=4	N=4	N=4	N=2	N=2	N=2	N=2	N=2

Supplemental Table S3 (continued): Complete data set for cell wall composition prior and following pretreatment, mass yields following pretreatments, quantified properties, and hydrolysis yields.

					Solubilized by Pretreatment				
	Initial Glucan	Final Glucan	Mass Yield	Ash	Glucan	Xylan	Lignin	pCA	FA
Name	$(g\ g^{-1})$	$(g\ g^{-1})$	$(g\ g^{-1})$	$(g\ g^{-1})$	$(g\ g^{-1})$	$(g\ g^{-1})$	$(g\ g^{-1})$	$(g\ mg^{-1})$	$(g\ mg^{-1})$
52220	0.322	0.387	0.700	0.026	0.051	0.015	0.121	4.79	4.90
A659	0.336	0.424	0.725	0.004	0.029	-0.010	0.074	6.82	5.72
B14A	0.350	0.415	0.725	0.004	0.048	-0.001	0.099	2.92	3.70
B47	0.326	0.458	0.737	0.010	-0.012	-0.018	0.120	7.19	5.91
B66	0.339	0.428	0.734	0.010	0.025	-0.021	0.123	7.63	5.12
B7	0.338	0.406	0.686	0.018	0.060	0.026	0.057	6.67	4.95
CH157	0.325	0.426	0.752	0.017	0.004	-0.011	0.132	5.74	5.91
F431	0.323	0.424	0.764	0.013	-0.001	-0.017	0.108	5.85	6.04
HUAN YAO	0.349	0.374	0.674	0.027	0.097	0.081	0.045	4.83	5.15
INB 101LFY/LFY (A632xM16S5)	0.329	0.437	0.763	0.036	-0.005	-0.009	0.100	5.18	6.71
Ky226	0.340	0.419	0.748	0.013	0.026	-0.011	0.093	7.97	5.80
LH143	0.326	0.397	0.681	0.007	0.056	0.082	0.065	5.11	4.66
LP1NR HT	0.340	0.422	0.748	0.005	0.024	0.061	0.110	7.05	6.25
Mo5	0.333	0.416	0.723	0.030	0.033	-0.015	0.107	5.35	5.36
N215	0.335	0.387	0.683	0.019	0.071	0.043	0.046	6.44	5.20
NC290A	0.324	0.433	0.698	0.029	0.022	-0.003	0.106	6.96	6.49
NC368	0.331	0.376	0.730	0.009	0.056	0.033	0.031	6.96	7.24
NK807	0.332	0.412	0.710	0.026	0.039	-0.025	0.099	6.11	5.02
No.380	0.299	0.378	0.718	0.023	0.028	-0.024	0.080	5.42	4.70
Oh7B	0.340	0.421	0.695	0.024	0.047	-0.005	0.120	7.34	6.10
PHG86	0.337	0.442	0.743	0.004	0.008	-0.012	0.124	5.22	6.94
W611S	0.330	0.414	0.693	0.012	0.043	-0.018	0.072	4.13	3.87
W64A	0.326	0.409	0.763	0.005	0.014	-0.011	0.106	7.51	6.96
W64A[7] bm4 S8	0.334	0.434	0.682	0.033	0.038	0.008	0.111	5.43	5.93
W64A[8]bm2 S6	0.332	0.381	0.741	0.032	0.050	0.042	0.051	5.31	4.21
Wf9	0.321	0.397	0.648	0.033	0.064	0.047	0.097	5.76	6.44
YE4	0.336	0.440	0.686	0.024	0.035	-0.003	0.078	2.71	3.36

	Error +/-	Error +/-	Error +/-	Error +/-
52220	0.026	0.001	0.003	0.005
A659	0.001	0.003	0.000	0.000
B14A	0.014	0.011	0.014	0.002
B47	0.008	0.012	0.004	0.002
B66	0.003	0.002	0.009	0.004
B7	0.013	0.003	0.000	0.004
CH157	0.001	0.001	0.002	0.001
F431	0.006	0.009	0.003	0.001
HUAN YAO	0.012	0.008	0.003	0.001
INB 101LFY/LFY (A632xM16S5)	0.013	0.002	0.009	0.002
Ky226	0.001	0.002	0.001	0.003
LH143	0.006	0.005	0.004	0.002
LP1NR HT	0.009	0.008	0.001	0.002
Mo5	0.014	0.003	0.006	0.002
N215	0.002	0.001	0.004	0.003
NC290A	0.006	0.008	0.018	0.003
NC368	0.000	0.000	0.007	0.002
NK807	0.008	0.005	0.003	0.002
No.380	0.009	0.023	0.002	0.008
Oh7B	0.004	0.005	0.001	0.004
PHG86	0.012	0.025	0.002	0.002
W611S	0.003	0.012	0.007	0.001
W64A	0.003	0.012	0.000	0.002
W64A[7] bm4 S8	0.008	0.029	0.002	0.005
W64A[8]bm2 S6	0.004	0.003	0.001	0.000
Wf9	0.004	0.021	0.011	0.001
YE4	0.008	0.008	0.009	0.003
Replicates:	N=2	N=2	N=2	N=2
